# Genetic interactions between the hedgehog co-receptors *Gas1* and *Boc* regulate cell proliferation during murine palatogenesis

**DOI:** 10.18632/oncotarget.13011

**Published:** 2016-11-02

**Authors:** Guilherme M. Xavier, Maisa Seppala, Spyridon N. Papageorgiou, Chen-Ming Fan, Martyn T. Cobourne

**Affiliations:** ^1^ Department of Craniofacial Development and Stem Cell Biology, King's College London Dental Institute, Guy's Hospital, SE1 9RT, London, UK; ^2^ Department of Orthodontics, King's College London Dental Institute, Guy's Hospital, SE1 9RT, London, UK; ^3^ Department of Orthodontics, School of Dentistry, University of Bonn, 53111, Bonn, Germany; ^4^ Department of Oral Technology, School of Dentistry, University of Bonn, 53111, Bonn, Germany; ^5^ Department of Embryology, Carnegie Institution of Washington, Baltimore, MD 21218, USA

**Keywords:** Shh signaling, palatogenesis, Gas1, Boc, cleft palate

## Abstract

Abnormal regulation of Sonic hedgehog (Shh) signaling has been described in a variety of human cancers and developmental anomalies, which highlights the essential role of this signaling molecule in cell cycle regulation and embryonic development. Gas1 and Boc are membrane co-receptors for Shh, which demonstrate overlapping domains of expression in the early face. This study aims to investigate potential interactions between these co-receptors during formation of the secondary palate. Mice with targeted mutation in *Gas1* and *Boc* were used to generate *Gas1*; *Boc* compound mutants. The expression of key Hedgehog signaling family members was examined in detail during palatogenesis via radioactive *in situ* hybridization. Morphometric analysis involved computational quantification of BrdU-labeling and cell packing; whilst TUNEL staining was used to assay cell death. Ablation of *Boc* in a *Gas1* mutant background leads to reduced Shh activity in the palatal shelves and an increase in the penetrance and severity of cleft palate, associated with failed elevation, increased proliferation and reduced cell death. Our findings suggest a dual requirement for *Boc* and *Gas1* during early development of the palate, mediating cell cycle regulation during growth and subsequent fusion of the palatal shelves.

## INTRODUCTION

**Figure 1:**
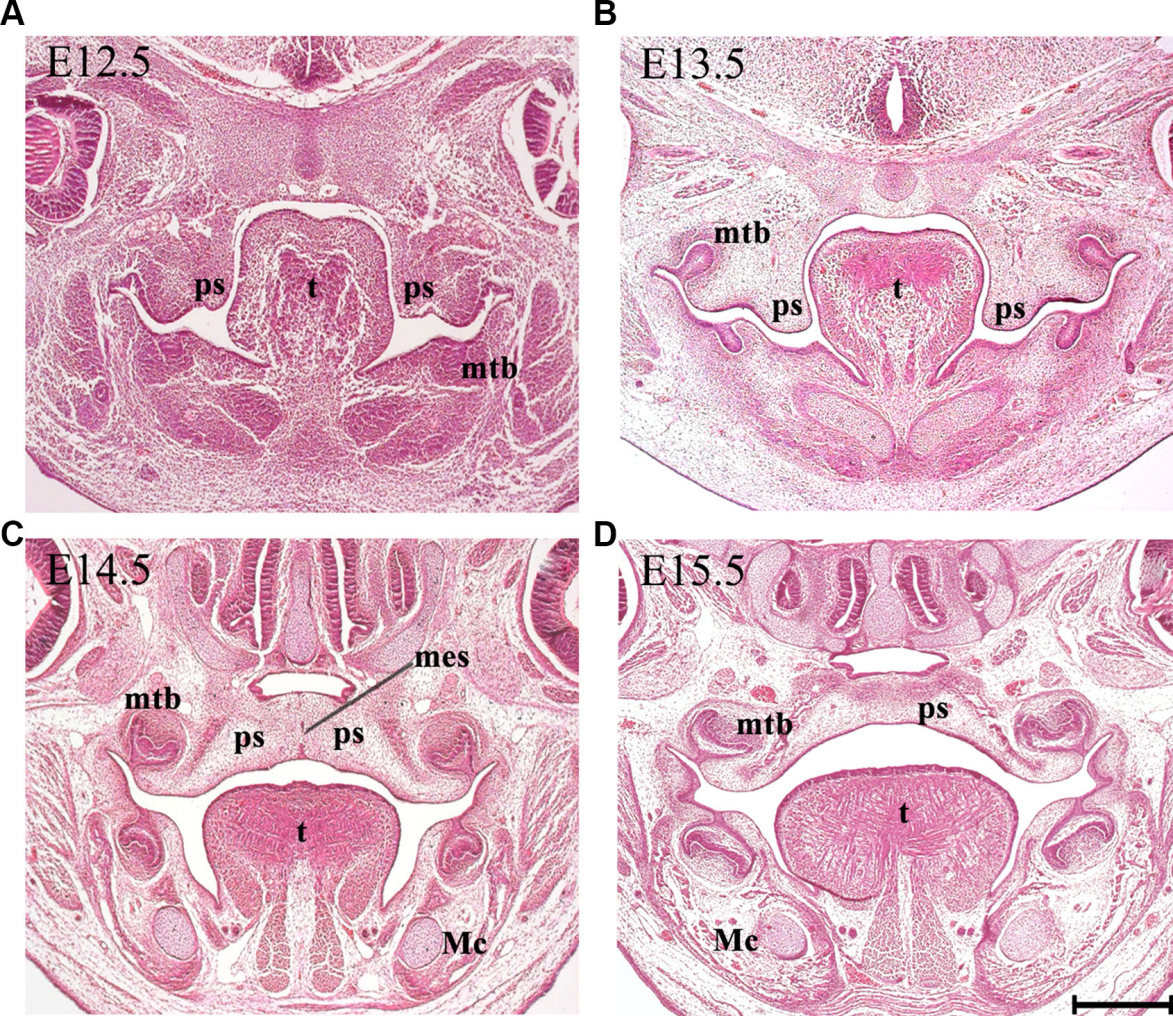
Palatogenesis in the mouse Representative histological frontal sections from the middle region of the developing palate at each indicated stage. (**A**) The secondary palate arises as paired outgrowths. (**B**) The PS initially grow vertically flanking the developing tongue. (**C**) The PS reorient to the horizontal position above the dorsum of the tongue in a process known as palatal shelf elevation. The fusion of palatal shelves involves the formation of a MES. (**D**) Subsequent disintegration of the MES allows mesenchymal confluence. Mc, Meckel's cartilage; mes, medial epithelium seam; mtb, molar tooth bud; ps, palatal shelf; t, tongue. Scale bar in D = 200 μm for A–D.

Development of the mammalian secondary palate is a complex process, which requires a coordinated network of molecular and cellular events to produce appropriate growth, elevation and fusion of the constituent palatal shelves [1–3]. In humans, palatogenesis occurs relatively early in development, taking place between 5 and 12 weeks of intrauterine life [4]. In the mouse, this process is remarkably similar to that in the human, but occurs more rapidly between embryonic stages (E) 10.5 and E15.5 [5]. Formation of the secondary palate begins with the appearance of two outgrowths from the maxillary process (palatal shelves, PS), which grow vertically to flank the lateral borders of the developing tongue (Figure 1B) [6, 7]. The PS subsequently elevate to a horizontal position above the tongue, which itself descends to help facilitate this process. Following elevation, medial growth of the paired PS towards the midline results in contact and then fusion with each other. During this stage, a transient medial epithelial seam (MES) is generated from the adhered epithelia [8, 9], which progressively disappears as midline confluence is achieved. The secondary PS also fuse with the primary palate anteriorly at the incisive foramen and complete confluence is usually observed around the twelfth week of human embryogenesis (E15.5 in mice) (Figure 1) [4, 8].

The Hedgehog (Hh) family of signaling molecules have emerged as major contributors to the developmental process in a wide variety of organisms, coordinating cell proliferation, survival and differentiation in multiple tissues [10–13]. Dysfunction of Hh signaling underlies a number of human developmental abnormalities and diseases, making it an important therapeutic target [10]. More specifically, genetic defects in the pathway can cause Holoprosencephaly (HPE) [14] or complex genetic diseases, such as Pallister–Hall syndrome [15] and Basal Cell Nevus Syndrome (BCNS) [16–18]. The Hh signaling pathway can undergo aberrant activation through the overexpression of Hh ligands, loss of receptor and co-receptor function or dysregulation of downstream transcription factors. All these aberrations in Hh signaling have been implicated in the initiation and progression of multiple cancer types, including breast, prostate, hepatocellular, pancreatic and brain cancers [11]. Sonic hedgehog (Shh) is the most comprehensively studied member of the Hh family [10] with the secreted ligand binding the primary Patched-1 (Ptch1) receptor to effect signal transduction [19, 20]. In the absence of ligand, Ptch1 acts as a ligand-independent inhibitor of the transmembrane protein Smoothened (Smo), a positive regulator of the pathway [10, 21, 22]. This regulation of Shh activity takes place in the primary cilium, by an as yet undefined mechanism [23–26]. Once the repression exerted by Ptch1 is released by Shh binding, increased ciliary levels of Smo lead to active transcription of Gli (Glioma-associated oncogene family members) transcription factors, through binding of specific consensus sequences located in the promoter region of target genes [10, 21]. More recently, the complexity of Shh signal regulation has become further evident as new proteins involved in modulating the pathway have been uncovered [21]. Among these, Growth arrest-specific 1 (Gas1) [27, 28], Cell-adhesion-molecule-related/downregulated by oncogenes (Cdon) [29, 30] and Biregional Cdon-binding protein (Boc) have been established as essential co-receptors that promote Shh signal transduction within a number of developmental contexts [31, 32]. Interestingly, some mutations causing HPE impair the palmitate-dependent interaction between Shh and Ptch1 [33, 34]. This interaction is also abolished in the BCNS, a congenital predisposition to cancers driven by hyperactive Hh signaling, such as basal cell carcinoma and medulloblastoma [34]. Not surprisingly, the features of HPE and aggressive basal-cell carcinomas have been previously reported in the same individual [35].

Gas1 is a N-glycosylated glycosylphosphatidyl inositol (GPI)-linked plasma membrane protein originally isolated via differential screening of fibroblasts maintained under growth arrest [36, 37]. Subsequently, *GAS1* was mapped to human chromosome 9q21.3-22.1 and established as a negative cell cycle regulator and tumor suppressor [38]. The first link between Hh signaling and *Gas1* was established through immunoprecipitation assays demonstrating Gas1 as capable of binding Shh and reducing its action [39]. However, subsequent *in vivo* studies have argued against these initial *in vitro* observations [27, 28, 40, 41]. Analysis of *Gas1* mutant mice have demonstrated malformations characteristic of *Shh* loss-of-function, including micropthalmia [42], HPE [27, 28], axon guidance deficiency and neural tube patterning defects [40, 41]. Moreover, depletion of *Shh* dosage in a *Gas1* mutant background leads to even more severe developmental defects [40]. These correlations and genetic interactions support the view that *Gas1* is a positive component of the Shh signaling pathway [27, 28, 40]. *Boc* was identified via screening of a human fetal brain cDNA library using a rat Cdon cDNA probe [43]. Biochemical analysis depicts Boc with a single transmembrane domain and four immunoglobulin like loops plus three fibronectin type III (FNIII) repeats in its ectodomain [43, 44]. *BOC* localizes to the plus strand of human chromosome 3q13.2 [45]. A study on the guidance of commissural axons in mice provided evidence to correlate *Boc* and Shh signaling [46]. Boc was shown to act as a receptor, capable of interacting directly with Shh via its third FNIII repeat (FNIIIc) [46]. Moreover, immunopreciptation experiments demonstrated that Boc can also physically bind to Ptch1 [31]. Interestingly, the presence of Shh does not alter the ability of Ptch1 to bind Boc, suggesting a constitutive interaction [31]. Recently, mutations affecting CDON disrupted its ability to interact with GAS1 and PTCH1, reinforcing the importance of these interactions for appropriate SHH signal reception. This mutation-induced disruption of interactions between SHH co-receptors has been shown to be a mechanism in HPE, a congenital anomaly associated with diminished Shh activity [47]. Taken together, these data have established the concept that these molecules can act as Hh co-receptors [32].

*Shh* transcriptional activity is detected in epithelium of the developing PS [48, 49] and the ligand plays a key role in mediating palatal outgrowth and patterning through an interaction with Fgf10 in the underlying mesenchyme [50]. Shh is also involved in a further regulatory feedback loop between epithelium and mesenchyme during growth of the PS, interacting with Bmp4 and Msx1 to induce proliferation in the mesenchyme [51]. Shh is also able to promote cell proliferation in the palatal mesenchyme via the activation of additional transcription factors, including Foxf1a, Foxf2 and Osr2 [52, 53]. More recently, tissue-specific deletion of *Pax9* from mesenchyme of the PS has been shown to indirectly regulate *Shh* expression in the adjacent epithelium and downregulate key targets in the mesenchyme (*Bmp4*, *Fgf10* and *Msx1*), placing *Pax9* upstream of this complex gene network [54, 55]. *Gas1^−/−^* mice also demonstrate cleft of the secondary palate (CP) with 50% penetrance, which is associated with reduced Shh signal transduction [28]. We have previously demonstrated that fine-tuning of Shh transduction is also crucial for PS fusion. The PS of transgenic mice overexpressing *Shh* in the PS epithelium under control of a Keratin-14 promotor (K14-*Shh*) demonstrate reduced cell death in the MEE, which prevents PS fusion [56]. Collectively, these findings highlight the importance of undisturbed Shh signaling during the events underlying normal palatogenesis.

There are currently over one thousand identified loci associated with orofacial clefting [57], but only around half of these have a defined molecular basis [58]. A precise integration between cell cycle regulation and cell-type specification is required during embryogenesis to direct the appropriate formation and function of each tissue. Gas1 and Boc have been shown to be key for harmonious integration of these two programs [31, 59–61]. Furthermore, disruption of *Gas1* and *Boc* has highlighted their importance in human diseases, including cancer [59, 62–64]. In the present investigation, we aim to further elucidate potential interactions between *Gas1* and *Boc* during cell cycle regulation in the developing palate. Significantly, ablation of *Boc* in a *Gas1* mutant background led to reduced Shh activity in the PS and increased severity of the CP phenotype. This was associated with failed PS elevation, increased mesenchymal proliferation and reduced epithelial cell death. Our findings suggest a dual requirement for *Boc* and *Gas1* during early palatogenesis, mediating cell proliferation during growth and cell survival during subsequent PS fusion.

## RESULTS

### Normal expression of *Shh*, *Ptch1*, *Gas1* and *Boc* during secondary palate development

**Figure 2:**
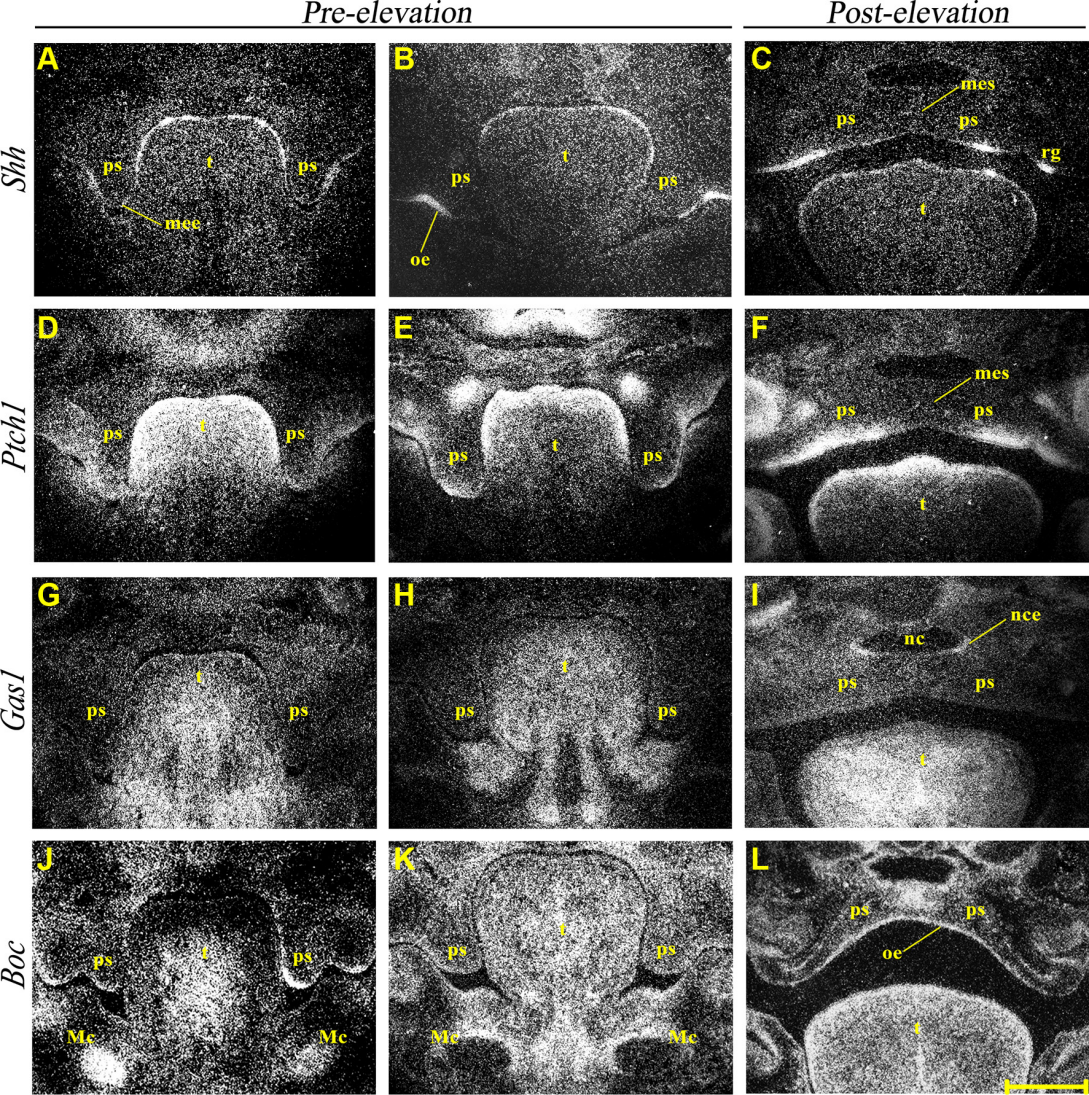
Normal expression of *Shh, Ptch1*, *Gas1* and *Boc* during palate development Radioactive *in situ* hybridization showing frontal sections of medial developing palate at pre (E12.5 **A**, **D**, **G**, **J** and E13.5 **B**, **E**, **H**, **K**) and post palatal shelf elevation (E14.5 **C**, **F**, **I**, and E15.5 **L**) and normal mRNA expression of *Shh* (A–C), *Ptch1* (D–F), *Gas1* (G–I) and *Boc* (J–L). Mc, Meckel's cartilage; mee, medial edge epithelium; mes, medial epithelium seam; nc, nasal cavity; nce, nasal cavity epithelium; oe, oral epithelium; ps, palatal shelf; rg, rugae; t, tongue. Scale bar in L = 200 μm for A–L.

*Shh* transcriptional activity was observed in the developing rugae of the PS oral epithelium between E12.5-14.5 (Figure 2A–2C), with transient transcriptional activity also seen in the future MEE region at E12.5 (Figure 2A). Shh signaling was therefore active during growth and elevation of the PS and confirmed by the presence of strong *Ptch1* expression in condensed mesenchyme adjacent to regions of *Shh* expression (Figure 2D–2F). However, *Ptch1* expression was not observed in the MES during fusion (Figure 2F). *Gas1* showed widespread expression within PS mesenchyme during growth of these structures in regions adjacent to those expressing *Ptch1* (Figure 2G–2I). Interestingly, *Gas1* was also upregulated in nasal epithelium of the PS following fusion (Figure 2I). In contrast, *Boc* showed diffuse low-level transcription in PS mesenchyme but strong expression within the epithelium at E12.5 (Figure 2J). Although the epithelial expression was somewhat downregulated at E13.5 (Figure 2K), transcripts were still observed in the mesenchyme. Following PS elevation and fusion, *Boc* transcriptional activity was detected throughout the oral palatal epithelium and within the region of the MES (Figure 2L).

### Interactions between *Gas1* and *Boc* during palatogenesis

**Figure 3:**
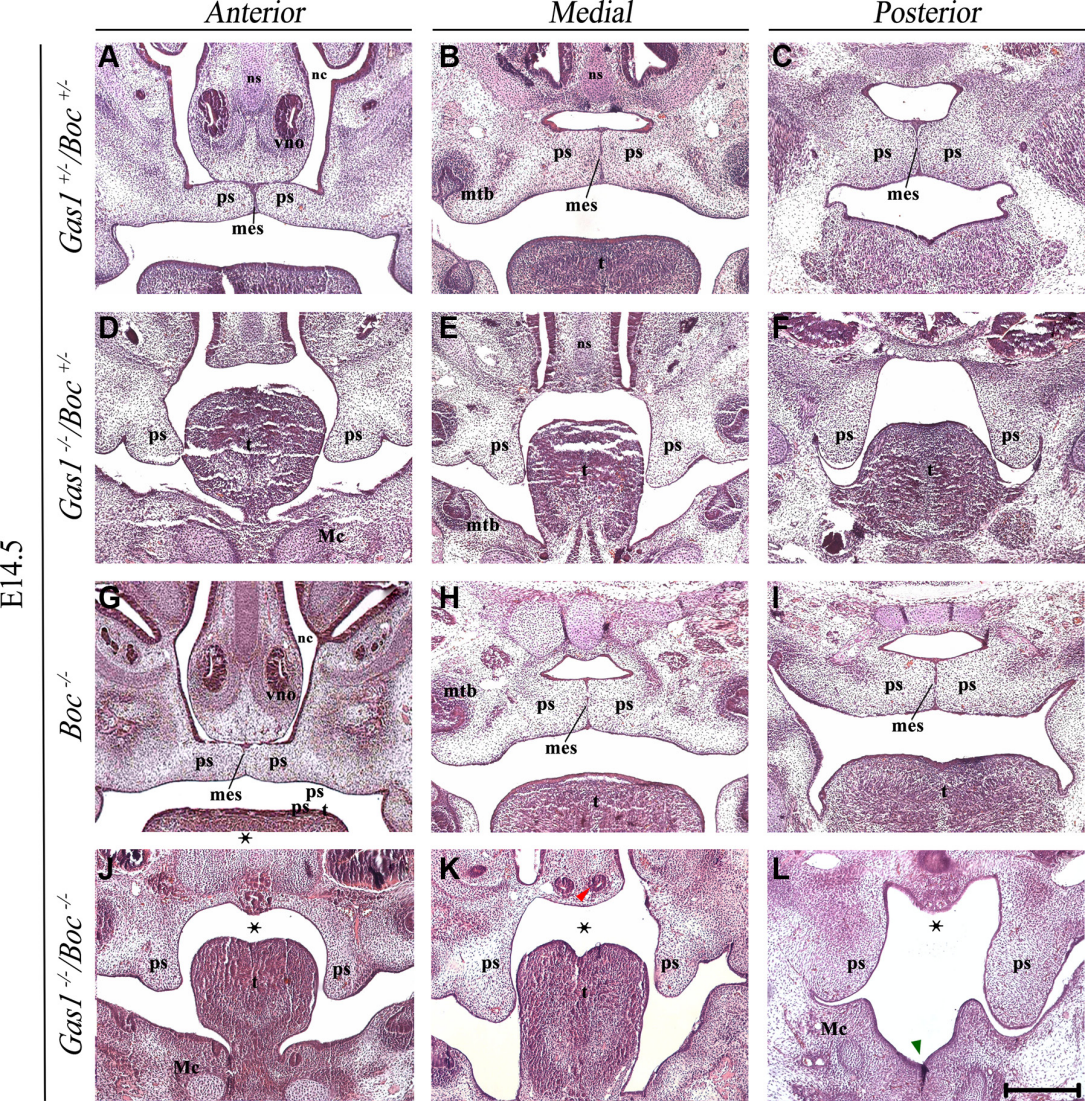
Histological phenotype of *Gas1^+/^*^−^*; Boc^+/^*^−^*, Gas1*^−*/*−^, *Boc^+/^*^−^, *Boc*^−*/*−^ and *Gas1*^−*/*−^*; Boc*^−*/*−^ palate Frontal sections of H&E stained E14.5 embryos through the anterior, medial and posterior palate. *Gas1^+/^*^−^*; Boc^+/^*^−^ (**A**–**C**), *Gas1^−/−^;Boc^+/^*^−^ (**D**–**F**), *Boc*^−*/*−^ (**G**–**I**) and *Gas1*^−*/*−^*; Boc*^−*/*−^ (**J**–**L**). The midline clefting within the posterior third of the tongue in the *Gas1*^−*/*−^*; Boc*^−*/*−^ embryo is highlighted by the green arrowhead in L. Abnormal positioning of the vno is highlighted by the red arrowhead in K. The black asterisks (J-L) indicate the CP associated with a failure of palatal shelf elevation observed in *Gas1*^−*/*−^*; Boc*^−*/*−^ mice. Mc, Meckel's cartilage; mes, medial epithelium seam; mtb, molar tooth bud; nc, nasal cavity; ns, nasal septum; ps, palatal shelf; t, tongue; vno, vomeronasal organ. Scale bar in L = 200 μm for A–L.

**Figure 4:**
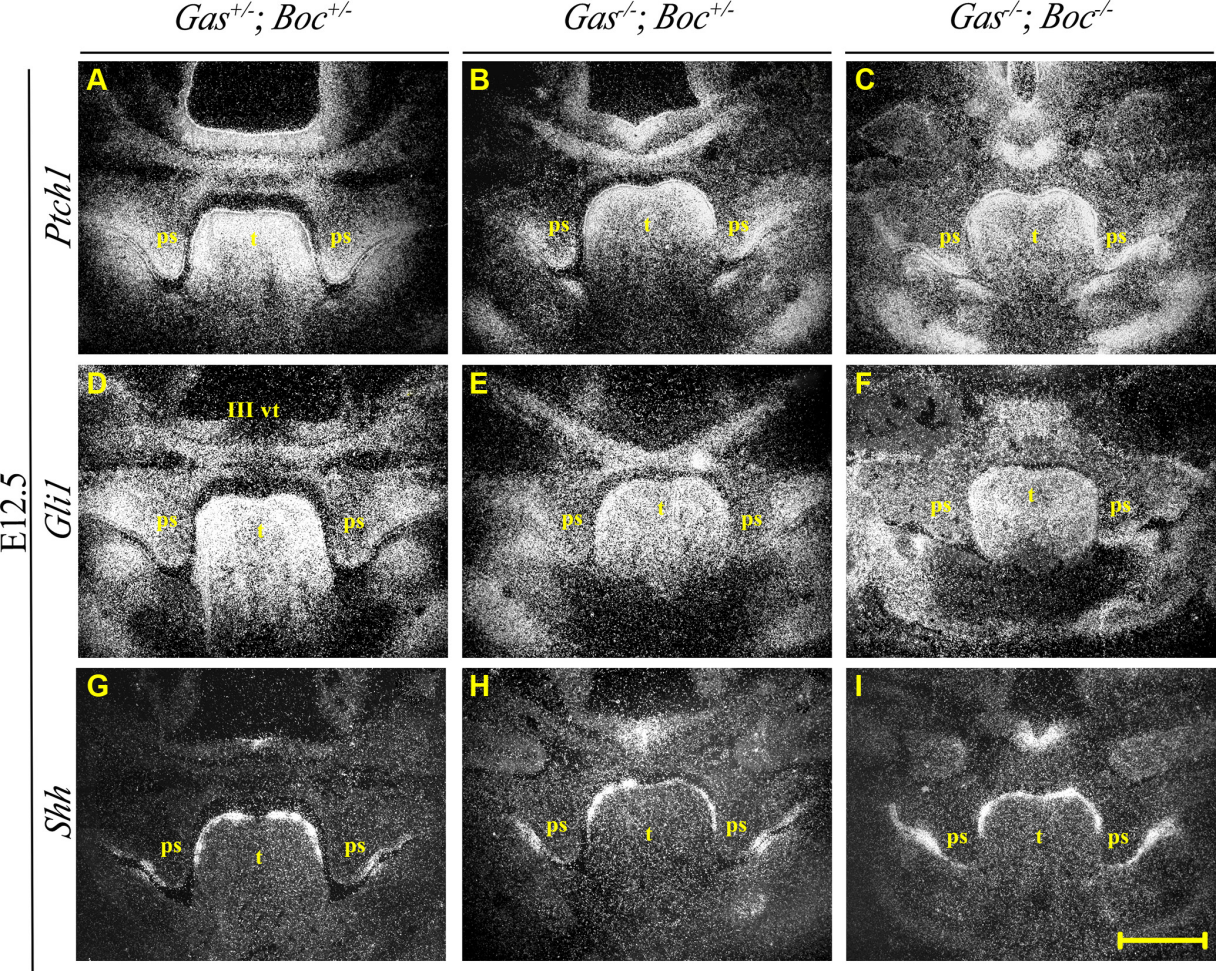
Expression of *Ptch1, Gli1* and *Shh* during palate development at E12.5 in *Gas1; Boc* compound mutants Radioactive *in situ* hybridization showing frontal sections of the medial developing palate at stage E12.5 of *Gas1^+/^*^−^*; Boc^+/^*^−^ (**A**, **D** and **G**), *Gas1*^−*/*−^*;Boc^+/^*^−^ (**B**, **E** and **H**) and *Gas1*^−*/*−^*; Boc*^−*/*−^ (**C**, **F** and **I**) mice. *Ptch1* (A–C), *Gli1* (D–F) and *Shh* (G–I). III vt, third ventricle; ps, palatal shelf; t, tongue. Scale bar in I = 200 μm for A–I.

Histological analysis of *Gas1^+/−^*; *Boc^+/−^* mice demonstrated a craniofacial midline comparable to wild type mice, with normal palatal development (Figure 3A–3C). *Gas1^−/−^*; *Boc^+/−^* mice exhibited microfom HPE, which has been previously described in *Gas1^−/−^* mice [28, 61] and includes CP with incomplete penetrance (Figure 3D–3F). In agreement with previous investigations, *Boc^−/−^* mice were viable, did not display any gross craniofacial phenotype and could not be distinguished from their wild type littermates [30, 31, 61]. Moreover, palatal development was not affected by an absence of *Boc* function (Figure 3G–3I). In contrast, *Gas1^−/−^*; *Boc^−/−^* compound mutant mice exhibited a fully penetrant CP associated with a failure of PS elevation above the tongue (Figure 3J–3L). In addition, an abnormally positioned vomeronasal organ was observed and a cleft tongue present in the pharyngeal region (Figure 3L). Significantly, the more severe craniofacial phenotype observed in *Gas1; Boc* compound mutants was associated with a reduction in expression levels of the Shh target genes *Ptch1* and *Gli1*, respectively (Figure 4A–4C; 4D–4F). However, transcriptional activity of *Shh* was seemingly unaltered when compared to control *Gas1^+/−^; Boc^+/−^* mice (Figure 4G–4I).

### Palatal shelf mesenchymal cell packing and proliferation indices in the absence of *Gas1* and *Boc* function

**Figure 5:**
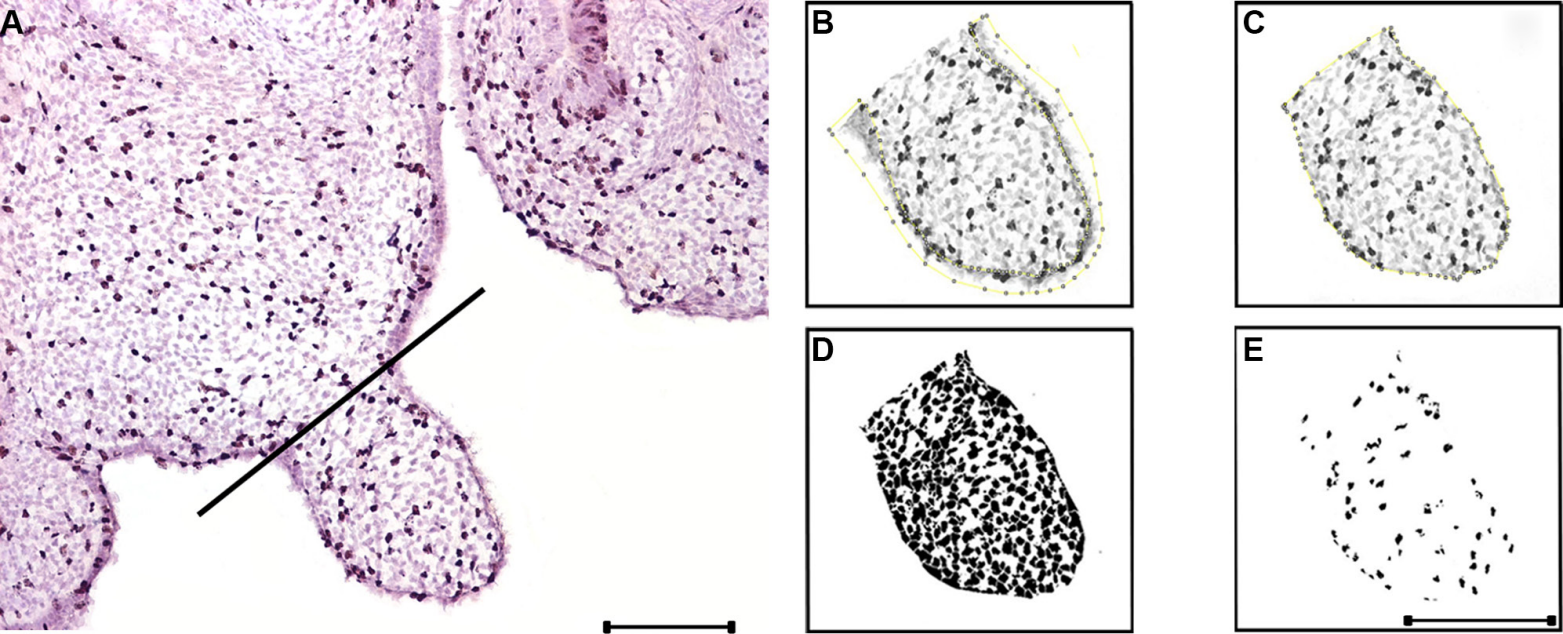
BrdU labeling and image analysis methodology (**A**) BrdU labeling. A perpendicular line from the palatal shelf ‘hinge’ to the opposite palatal surface delimitates the analysed area. (**B**) The epithelium is deleted. (**C**) The region of interest is delimitated, and subsequently measured. (**D**) Thresholding for the total cells within the region of interest; the watershed plugin is applied for segmentation and the total cell counting is obtained. (**E**) Thresholding for the BrdU positive cells within the region of interest; the watershed plugin is applied for segmentation and the positive cell counting is obtained. Scale bar in A = 200 μm for (A). Scale bar in E = 200 μm for (B–E).

**Figure 6:**
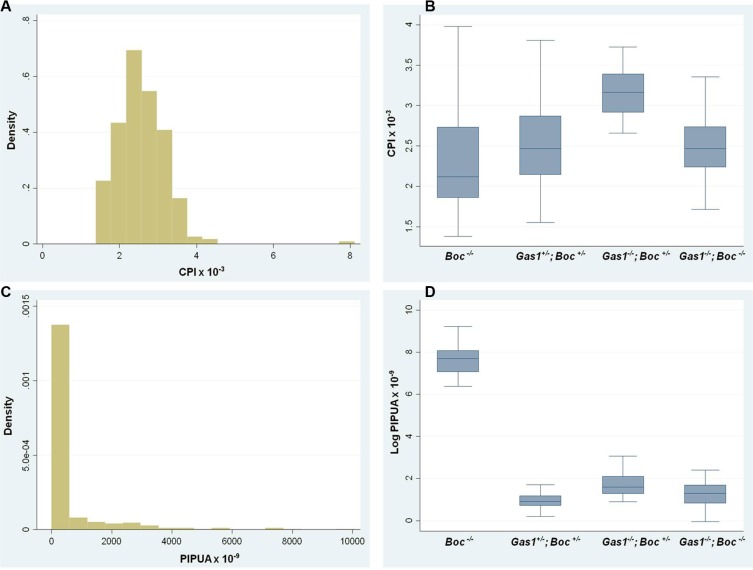
Cell packing and proliferation in the developing palate of *Gas1^+/^*^−^*; Boc^+/^*^−^*, Gas1*^−*/*−^, *Boc^+/^*^−^, *Boc*^−*/*−^ and *Gas1*^−*/*−^*; Boc*^−*/*−^ mice at E14.5 (**A**) Histogram for the CPI values indicates that the data is not normally distributed. (**B**) CPI box plots for the genotypes analysed. (**C**) Histogram for the PIPUA values indicates that the data is not normally distributed. (**D**) PIPUA box plots for the genotypes analysed. CPI, cell packing index; PIPUA, proliferation index per unit area

The phenotypic analysis of *Gas1* and *Boc* single and compound mutant mice was suggestive of a role for these co-receptors during the regulation of PS growth. We therefore analysed the PS phenotype in these mutants at the cellular level, specifically focusing on the mesenchymal component Figure 5. In mesenchymal tissues, the extracellular matrix can contribute significantly to tissue volume, therefore we also incorporated a measure of cell spacing [65]. Specifically, we generated a cell packing index (CPI) and a proliferation index per unit area (PIPUA) within the PS using image segmentation to determine total and BrdU-positive cells within the mesenchyme [66]. A descriptive analysis of the CPI is shown in Table 1, containing the number of PS analysed for each genotype, the median, range and interquartile range. Kruskal-Wallis test revealed a statistical significant difference among the four genotypes analyzed (*p* < 0.001). Table 2 illustrates the Poisson regression analysis performed to evaluate CPI differences among the genotypes. *Post hoc* pairwise comparisons demonstrated that the only non-statistical significant result was the CPI difference between *Gas1^−/−^; Boc^−/−^* and *Gas1^+/−^; Boc^+/−^* PS (*p* = 0.636) (Figure 6B). In fact, the same median was observed for both groups (*Gas1^−/−^; Boc^−/−^* and *Gas1^+/−^; Boc^+/−^*) (Table 1). *Gas1^−/−^; Boc^+/−^* PS showed a higher CPI compared to control (*Gas1^+/−^; Boc^+/−^*); whereas *Boc^−/−^* PS showed the lowest CPI amongst genotypes (Figure 6B, Table 2). The CPI is a measure of cell density; that is the number of cells per region of interest. It then follows that upon comparison of two samples (e.g. *Gas1^+/−^; Boc^+/−^* versus *Gas1^−/−^; Boc^−/−^*) if the cell density is constant, any differences in the PIPUA denotes a true change in proliferation as a result of mutation in *Gas1* and *Boc* or genetic interaction. Analysis of the PIPUA revealed a statistical significant difference among the four genotypes (*p* < 0.001). Table 3 illustrates descriptive analysis for the PIPUA, containing the number of PS analysed for each genotype, the median, range and interquartile range. *Post hoc* pairwise comparisons among groups revealed a statistical significant difference between PIPUA amongst all genotypes, except for *Gas1^−/−^; Boc^−/−^* versus *Gas1^−/−^; Boc^+/−^* (Figure 6D). Table 4 illustrates the Poisson regression analysis performed to evaluate PIPUA differences among the genotypes. The PIPUA of *Boc^−/−^* PS showed the highest value (2612.54, *p* < 0.001), whilst *Gas1^−/−^; Boc^+/−^* and *Gas1^−/−^; Boc^−/−^* PS also demonstrated a higher PIPUA compared to control, but to a lesser extent (Table 4, Figure 6D).

**Table 1:** CPI descriptive analysis

Genotypes	N	Median	*Q1-Q3*	*IQR*	*Range*
***Boc^−/−^***	55	2.12	1.86–2.73	0.87	1.38–4.29
***Gas1^+/−^***;***Boc^+/−^***	127	2.47	2.15–2.87	0.72	1.55–3.81
***Gas1^−/−^***; ***Boc^+/−^***	44	3.16	2.92–3.39	0.47	1.47–4.44
***Gas1^−/−^***; ***Boc^−/−^***	65	2.47	2.24–2.73	0.49	1.72–8.11
**Overall**	291	2.51	2.15–2.96	0.81	1.38–8.11

N, number of PS; IQR, interquartile range.

**Table 2:** CPI poisson regression analysis

GLM Poisson regression				Pairwise comparisons (*p* values)				
Genotypes	Coef	95% CI	*P* value	Genotypes	*Boc^−/−^*	*Gas1^+/−^*; *Boc^+/−^*	*Gas1^−/−^*; *Boc^+/−^*	*Gas1^−/−^*; *Boc^−/−^*
***Boc^−/−^***	−0.21	−0.41, −0.03	0.025	***Boc^−/−^***	_	_	_	_
***Gas1^+/−^***; ***Boc^+/−^***	Baseline	_	_	***Gas1^+/−^***; ***Boc^+/−^***	0.025	_	_	_
***Gas1^−/−^***; ***Boc^+/−^***	0.61	0.45,0.77	< 0.001	***Gas1^−/−^***; ***Boc^+/−^***	< 0.001	< 0.001	_	_
***Gas1^−/−^***; ***Boc^−/−^***	0.05	−0.16,0.26	0.636	***Gas1^−/−^***; ***Boc^+/−^***	0.040	0.636	< 0.001	_

Coef, Poisson regression coefficients for the model; CI, confidence interval; GLM, generalized linear models.

**Table 3:** PIPUA descriptive analysis

Genotypes	N	Median	*Q1-Q3*	*IQR*	*Range*
***Boc^−/−^***	55	2175.3	1170.19–3184.81	2014.62	585.13–10055.89
***Gas1^+/−^***; ***Boc^+/−^***	127	2.51	2.09–3.24	1.15	1.23–8.17
***Gas1^−/−^***; ***Boc^+/−^***	44	4.90	3.63–8.15	4.52	2.45–21.18
***Gas1^−/−^***; ***Boc^−/−^***	65	3.63	2.30–5.38	3.09	0.96–40.18
**Overall**	291	3.52	2.39–8.17	5.78	0.96–10055.99

N, number of PS; IQR, interquartile range.

**Table 4:** PIPUA poisson regression analysis

GLM Poisson regression				Pairwise comparisons (*p* values)				
Genotypes	Coef	95% CI	*P* value	Genotypes	*Boc^−/−^*	*Gas1^+/−^*; *Boc^−/−^*	*Gas1^−/−^*; *Boc^−/−^*	*Gas1^−/−^*; *Boc^−/−^*
***Boc^−/−^***	2612.54	2084.14,3140.95	< 0.001	***Boc^−/−^***	_	_	_	_
***Gas1^+/−^***; ***Boc^+/−^***	Baseline	_	_	***Gas1^+/−^***; ***Boc^+/−^***	< 0.001	_	_	_
***Gas1^−/−^***; ***Boc^+/−^***	3.48	2.40,3.83	< 0.001	***Gas1^−/−^***; ***Boc^+/−^***	< 0.001	< 0.001	_	_
***Gas1^−/−^***; ***Boc^−/−^***	2.38	0.92,3.83	0.001	***Gas1^−/−^***; ***Boc^−/−^***	< 0.001	0.001	0.228	_

Coef, Poisson regression coefficients for the model; CI, confidence interval; GLM, generalized linear models.

### Palatal shelf cell survival in the absence of *Gas1* and *Boc* function

**Figure 7:**
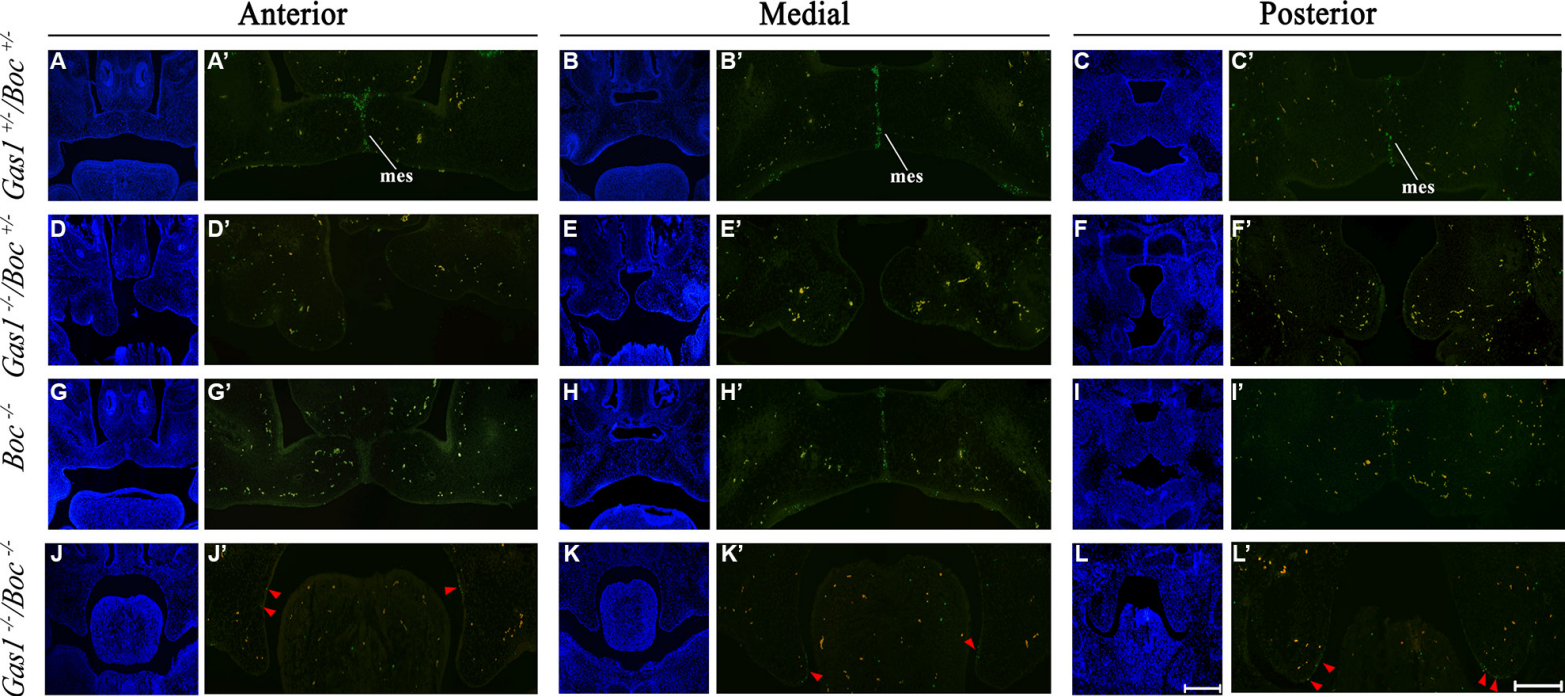
Cell survival in the developing palate of *Gas1^+/^*^−^*; Boc^+/^*^−^*, Gas1*^−*/*−^; *Boc^+/^*^−^, *Boc*^−*/*−^ and *Gas1*^−*/*−^*; Boc*^−*/*−^ mice at E14.5 Frontal sections through the anterior, medial and posterior regions of the developing palate. *Gas1^+/^*^−^*; Boc^+/^*^−^ (**A**–**C**, A'-C'), *Gas1*^−*/*−^*;Boc^+/^*^−^ (**D**–**F**, D'-F'), *Boc*^−*/*−^ (**G**–**I**, G'-I') and *Gas1*^−*/*−^*; Boc*^−*/*−^ (**J**–**L**, J'-L'). 4′, 6-diamidino-2-phenylindole (DAPI) staining (A–L) to visualize cell nuclei and tissue architecture. Merged images (A'-L') used to distinguish between TUNEL-positive cells (green fluorescence) and background staining (orange). Red arrowheads in (J'-L') highlight the TUNEL-positive cells. Scale bar in L = 200 μm for (A–L). mes, medial epithelial seam. Scale bar in L' = 100 μm for (A'-L').

Regression of the MES is an important step during palatogenesis and contributes to formation of a confluent secondary palate [5]. Programmed cell death (apoptosis) is one of the proposed mechanisms involved in mediating MES degeneration [58, 67]. In the present study, we assayed the presence of apoptotic cells using TUNEL assays. Interestingly, we found similar levels of apoptosis in the anterior, medial and posterior sections of *Boc^−/−^* PS when compared to corresponding sections of *Gas^+/−^; Boc^+/−^* PS (Figure 7G'-I'; A'-C', respectively). Conversely, the levels of cell death within *Gas^−/−^; Boc^+/−^* (Figure 7D'–7F') and *Gas^−/−^; Boc^−/−^* PS was reduced in relation to *Gas^+/−^; Boc^+/−^.*

## DISCUSSION

Understanding the role of Shh during palatogenesis is important because of the key regulatory role this signaling protein plays during development of this structure [68]. *Gas1* and *Boc* are now established as essential Shh co-receptors during development and are required for regulating Shh-mediated cell proliferation in other regions of the embryo [31, 32]. Shh pathway components demonstrate distinct regional expression in the PS during development [28, 49, 50] and here we have shown non-overlapping domains between *Ptch1*, *Gas1* and *Boc* in the epithelium and mesenchyme during palatogenesis. Recent evidence suggests that the co-receptor function exerted by Gas1 and Boc in combination with Ptch1 is unlikely to involve all three molecules in the same complex [31]. The observed *Boc* expression pattern shows evidence for redundancy with *Gas1* in the palate, as previously demonstrated in other regions of the developing embryo, such as the neural tube and heart [32].

Ablation of *Boc* activity in a *Gas1* mutant background leads to a unique form of HPE [61]. Although *Boc^−/−^* mice were viable and fertile with no overt embryonic phenotype (Figure 3G–3I), *Gas1^−/−^; Boc^−/−^* embryos show defects not previously observed in mice lacking *Gas1* activity [28, 61]. Of relevance to palatal development, *Gas1; Boc* compound mutants exhibited a fully penetrant CP, associated with failed elevation of the PS. Other phenotypes included clefting of the posterior tongue and abnormal positioning of the vomeronasal organ. These characteristics correlate with a reduction of Shh signaling, which seems more drastically affected in *Gas1; Boc* compound mutants (Figure 4). Similarly, in the context of limb development, a more severe defect in digit patterning and specification is observed in *Gas1; Boc* compound mutants [32]. In addition, Gas1 and Boc in conjunction with Cdon have recently been shown to modulate the levels of Hh-responsiveness in the pathogenesis of pancreatic cancer. When all three co-receptors are ablated intra-tumoral proliferation is reduced, highlighting the importance of combined mutagenesis screens when describing pathway function [62].

Whole population cell analysis of the PS epithelium has highlighted the importance of considering factors other than cell proliferation in isolation when evaluating tissue growth [65]. Here we focused on the mesenchyme, as elevation and growth of the PS is likely to be driven by changes in the mesenchymal stroma [8]. In order to determine the effects of targeted mutations in *Gas1* and *Boc* on the developing PS, BrdU and TUNEL assays were performed. A CPI and a PIPUA were implemented in order to generate an unbiased proliferation map of the entire PS mesenchyme (rather than randomly selecting isolated areas of tissue) taking into account the interdependent relationship of the two quantities [28, 56, 65, 69]. However, *Boc^−/−^* PS presented a decreased cell density (CPI) and increased proliferation (PIPUA) in comparison to control (*Gas^+/−^; Boc^+/−^* mice). These seemingly conflicting results can be explained by two possible mechanisms: (1) either an increased compensatory apoptosis, resulting in a net reduction in cell number or (2) an increase in the average distance between cells as a result of an increase in overall tissue size. The former hypothesis can be excluded following the apoptosis analysis, which demonstrated cell death present primarily in the epithelium at levels similar to those observed in *Gas^+/−^; Boc^+/−^* PS (Figure 7). Similar results in terms of cell death have also been observed in a different context (cerebellar granule neuron progenitors), where *Boc* ablation does not affect apoptosis [31]. As no overt differences were observed in overall tissue size, the alternative hypothesis would require further analysis (whereby cell distances are measured directly) in order to determine the precise causation of a cell proliferation increase with concomitant cell packing decrease. In *Gas^−/−^; Boc^+/−^* PS there was increased PIPUA accompanied by an increased CPI. This suggests a more straightforward relationship between *Gas1* and proliferation, whereby *Gas1* acts as a negative regulator of cell proliferation in the PS mesenchyme. This is in agreement with other studies demonstrating that *Gas1* is capable of initiating apoptosis and inhibiting proliferation [70, 71]. Interestingly, *Gas1* exerts similar functions in oncogenesis [63, 72]. Gas1 activity detains tumour growth by inhibiting the proliferation of breast cancer cells [63] and has been reported to play the same mechanistic role in a variety of other cancers; such as colorectal carcinoma [72], papillary thyroid carcinoma [64] and glioma [70]. In *Gas^−/−^; Boc^−/−^* PS the CPI was restored to levels observed in *Gas^+/−^; Boc^+/−^* suggesting that the two genes have opposing roles in regulating cell density. However, their relationship with respect to proliferation regulation appears to be more complex and non-synergistic, as demonstrated by the (significantly higher) PIPUA observed in the *Gas^−/−^; Boc^−/−^* PS. Although both genes seem to be negative regulators of proliferation in this developmental context, it is highly suggestive that additional regulators play a role in this network. Moreover, higher CPI and PIPUA are not necessarily an indication of aberrant palatogenesis, as observed in *Boc^−/−^* embryos. Therefore, it is reasonable to speculate that the HPE midline facial anomalies present in *Gas1^−/−^; Boc^−/−^* [61] could play an important role in the CP phenotype observed in these mice.

We have excluded tissue packing changes as a potential cellular mechanism underlying the *Gas1; Boc* mutant PS phenotype. Histological analysis demonstrated that the *Gas1^−/−^; Boc^−/−^* PS size are similar to that of *Gas^+/−^; Boc^+/−^*. Therefore, in order to further understand how the observed differences in proliferation contribute to the CP phenotype, a direct measure of the overall midfacial region of *Gas1^−/−^; Boc^−/−^* mice would be required. This may prove challenging to perform in plane section, because no account would be taken of cellular movements and rearrangements that might be taking place in the z- dimension [65, 69]. Alternative approaches might include three-dimensional and potentially live imaging, and cell tracking to encompass cellular rearrangements; these techniques would underpin our future studies. Recent reports of extensive cellular rearrangements in oral epithelia render this scenario plausible [73]. Similar experimental approaches could be adopted to further elucidate the links between genetic lesions and the cellular mechanistic defects underlying the CP phenotype. We have previously demonstrated that increased transduction of Shh signaling in the PS mesenchyme leads to reduced proliferation [56]. The results of the present study illustrate an opposite effect (increased PIPUA in *Gas1^−/−^; Boc^−/−^* mice) that correlates with reduced transcriptional activity of Shh signaling readouts. Furthermore, deletion of *Gas1* leads to reduced apoptosis in the PS. Although the CP phenotype in mice lacking *Gas1* [28] or in compound *Gas1; Boc* mutants is associated with PS that fail to elevate above the tongue; it is unlikely that the PS would fuse, as demonstrated by transgenic mice over-expressing Shh in the oral epithelium [56].

The results from this study further highlight the importance of Shh signaling in coordinating the process of palatogenesis. Hh family members are expressed at key stages during palate development [49, 56, 61]. Moreover, ablation of *Boc* in a *Gas1* mutant background leads to reduced transduction of Shh signaling. Morphometric analysis revealed that the more severe clefting phenotype observed in these mice was associated with higher proliferation levels and reduced apoptosis. Additional mRNA expression analysis of known mediators of palatal development may help to further define a gene network in developing palate. While systems approaches are important to elucidate the vast molecular network regulating complex developmental processes such as palatogenesis, understanding the role of individual genes implicated in cell regulation is also highly valuable. This study has directly addressed the role of two key Hh signaling components and their dual requirement for orchestrating palatogenesis. Similar studies addressing the roles of other key Hh components should eventually lead to a more complete picture of the genetic basis of midline development and how it relates to human syndromic disorders.

## MATERIALS AND METHODS

### Generation of *Gas1; Boc* compound mutant mice

All mice were housed and all experiments conducted in compliance with the approved protocols at King's College London, UK and the Carnegie Institution of Washington, USA. *Gas1^−/−^* mice were generated and maintained in a 129sv/C57BL6 mixed background and genotyped as previously described [27]. *Boc^−/−^* mice were generated and maintained in a CD1/129sv mixed background and genotyped as previously described [46]. *Gas1^+/−^* mice were crossed with *Boc^+/−^* mice, to generate *Gas1^−/−^*; *Boc^−/−^* compound mutants in a mixed background (129sv/C57BL/6/CD1). Timed-matings were set up such that noon of the day on which vaginal plugs were detected was considered as embryonic day (E) 0.5.

### Histological analysis

For histological analysis, embryos were fixed in 4% paraformaldehyde (PFA) at 4°C, dehydrated through a graded ethanol series, embedded in paraffin wax, sectioned at 7 mm and stained with haematoxylin and eosin (H&E).

### *In Situ* hybridization

Radioactive section *in situ* hybridization was carried out as previously described [74]. Dark-field images of sections were photographed using a Zeiss Axioscop microscope and montages constructed using Adobe Photoshop CS.

### Cell packing and proliferation index per unit area assays

A CPI was generated by dividing the total number of cells by the area of the region of interest. Assays for cell proliferation were carried out using a Zymed Bromodeoxyuridine (BrdU) Labeling and Detection Kit (Invitrogen), according to the manufacturer instructions. Mouse embryos were labeled with BrdU via intra-peritoneal injection into pregnant females (5 mg/100 g body weight) 2 hours prior to sacrifice. Slides were photographed using a Zeiss Axioscop microscope (Germany).

The imaging software package FIJI [66] was used to count BrdU-positive and total cells. Cells were counted in the mesenchyme of the anterior, medial and posterior palate. The lateral extent of the palate shelf was determined by drawing a perpendicular line from the “hinge” region to the opposite palatal surface [75]. Morphological segmentation of cells was performed using manual thresholding, followed by watersheding, to improve segmentation of closely neighboring cells [76]. Segmentation was performed twice, once for total and once for BrdU-positive cells. The palatal shelf area was measured by selecting the region of interest with the polygon selection tool. A proliferation index was first generated by dividing the number of positive cells by the total number of cells. The proliferation index per unit area (PIPUA) was generated by dividing the proliferation index by the region of interest. Due to the small numeric scale of the data and to make it more easily presentable, CPI was multiplied by 10^−3^ and PIPUA was multiplied by 10^−9^. The graph illustrating the PIPUA is in a logarithmic scale to facilitate visualization of the data.

### Apoptosis

Immunohistochemical detection of apoptotic cell death was carried out on histological sections using Terminal deoxynucleotidyl transferase-mediated deoxyUridine triPhosphate Nick End Labeling (TUNEL). TUNEL was carried out using an APOPTag^®^ Plus Fluorescein *In Situ* Apoptosis Detection Kit (Chemicon International) according to the manufacturer's instructions. Slides were photographed using a Zeiss Axioscop microscope (Germany).

### Statistical analysis

The assumption of normality for each variable was checked with with the Shapiro-Wilk test [77]. The assumption of homoscedascity was carried out with an information matrix test, according to Cameron and Trivedi [78]. As both the CPI and the PIPUA were not normally distributed (*p* < 0.001 from the Shapiro-Wilk test for both), the median and interquartile range (IQR) are reported as descriptive statistics. The Kruskal-Wallis test was used to test for differences in CPI and PIPUA among the four groups. Differences among the four groups were identified by calculating coefficients and the corresponding 95% confidence intervals (95% CI) through generalized linear regression models, only if the null hypothesis was rejected with the Kruskal-Wallis test, so as to reduce the risk of increased Type II error. According to inspection of the histograms and to model fit, a Poisson distribution was adopted for the models with calculation of robust standard errors to control for mild violation of underlying assumptions [79]. As *post hoc* pairwise comparisons among groups were performed only in case of a statistically significant Kruskal-Wallis test and these were of explorative nature, no P-value correction was applied. All statistical analyses were conducted with a 2-sided α of 5% in Stata version 12 (StataCorp LP, College Station, TX) with the macros swilk, kwallis, and glm.
